# Tracking Ovine Pulmonary Adenocarcinoma Development Using an Experimental Jaagsiekte Sheep Retrovirus Infection Model

**DOI:** 10.3390/genes15081019

**Published:** 2024-08-02

**Authors:** Chris Cousens, James Meehan, David Collie, Steven Wright, Ziyuan Chang, Helen Todd, Jo Moore, Lynn Grant, Carola R. Daniel, Peter Tennant, Adrian Ritchie, James Nixon, Chris Proudfoot, Stefano Guido, Helen Brown, Calum D. Gray, Tom J. MacGillivray, R. Eddie Clutton, Stephen N. Greenhalgh, Rachael Gregson, David J. Griffiths, James Spivey, Nicole Storer, Chad E. Eckert, Mark Gray

**Affiliations:** 1 Moredun Research Institute, Pentlands Science Park, Bush Loan, Penicuik EH26 0PZ, UK; chris.cousens@moredun.ac.uk (C.C.); helen.todd@moredun.ac.uk (H.T.); jo.moore@moredun.ac.uk (J.M.); david.griffiths@moredun.ac.uk (D.J.G.); 2 The Royal (Dick) School of Veterinary Studies and Roslin Institute, University of Edinburgh, Easter Bush, Roslin, Edinburgh EH25 9RG, UK; james.meehan@ed.ac.uk (J.M.); david.collie@roslin.ed.ac.uk (D.C.); steven.wright@ed.ac.uk (S.W.); zchang@exseed.ed.ac.uk (Z.C.); lynn.grant@ed.ac.uk (L.G.); carola.daniel@ed.ac.uk (C.R.D.); peter.tennant@roslin.ed.ac.uk (P.T.); aritchi3@exseed.ed.ac.uk (A.R.); james.nixon@roslin.ed.ac.uk (J.N.); chris.proudfoot@roslin.ed.ac.uk (C.P.); stefano.guido@ed.ac.uk (S.G.); helen.brown@roslin.ed.ac.uk (H.B.); e.clutton@ed.ac.uk (R.E.C.); stephen.greenhalgh@ed.ac.uk (S.N.G.); rachael.gregson@ed.ac.uk (R.G.); 3 Edinburgh Imaging Facility, Queen’s Medical Research Institute, University of Edinburgh, 47 Little France Crescent, Edinburgh EH16 4TJ, UK; calum.gray@ed.ac.uk; 4 Centre for Clinical Brain Sciences, College of Medicine and Veterinary Medicine, University of Edinburgh, Edinburgh, EH16 4SB, UK; t.j.macgillivray@ed.ac.uk; 5 Interventional Oncology, Johnson & Johnson Enterprise Innovation, Inc., One Johnson & Johnson Plaza, New Brunswick, NJ 08933, USA; jspivey4@its.jnj.com (J.S.); nstorer@its.jnj.com (N.S.); ceckert1@its.jnj.com (C.E.E.)

**Keywords:** ovine pulmonary adenocarcinoma, computed tomography, ultrasound, animal models

## Abstract

Ovine pulmonary adenocarcinoma (OPA) is an infectious, neoplastic lung disease of sheep that causes significant animal welfare and economic issues throughout the world. Understanding OPA pathogenesis is key to developing tools to control its impact. Central to this need is the availability of model systems that can monitor and track events after Jaagsiekte sheep retrovirus (JSRV) infection. Here, we report the development of an experimentally induced OPA model intended for this purpose. Using three different viral dose groups (low, intermediate and high), localised OPA tumour development was induced by bronchoscopic JSRV instillation into the segmental bronchus of the right cardiac lung lobe. Pre-clinical OPA diagnosis and tumour progression were monitored by monthly computed tomography (CT) imaging and trans-thoracic ultrasound scanning. Post mortem examination and immunohistochemistry confirmed OPA development in 89% of the JSRV-instilled animals. All three viral doses produced a range of OPA lesion types, including microscopic disease and gross tumours; however, larger lesions were more frequently identified in the low and intermediate viral groups. Overall, 31% of JSRV-infected sheep developed localised advanced lesions. Of the sheep that developed localised advanced lesions, tumour volume doubling times (calculated using thoracic CT 3D reconstructions) were 14.8 ± 2.1 days. The ability of ultrasound to track tumour development was compared against CT; the results indicated a strong significant association between paired CT and ultrasound measurements at each time point (R^2^ = 0.799, *p* < 0.0001). We believe that the range of OPA lesion types induced by this model replicates aspects of naturally occurring disease and will improve OPA research by providing novel insights into JSRV infectivity and OPA disease progression.

## 1. Introduction

Ovine pulmonary adenocarcinoma (OPA) is an infectious neoplastic lung disease of sheep. Caused by Jaagsiekte sheep retrovirus (JSRV), the disease passes between sheep largely through the inhalation of viral particles [1,2,3,4]. OPA has been identified in most sheep-producing countries, and with typical incidence levels of 1–5%, the disease causes significant economic and animal welfare issues [3,5,6,7]. Robust pre-clinical diagnostic tests, vaccines, or practice-changing policies for the control or eradication of OPA have yet to be developed. An experimental infection model that can be used to accurately monitor tumour development over time and allows for samples to be taken from defined stages of disease progression will be useful to advance the understanding of JSRV pathogenesis and lead to improvements in the control of OPA in sheep flocks.

Ex vivo and in vitro alternatives to animal models do exist, and indeed, immortalised cell lines are commonly used in this regard. However, the lack of an immortalised cell line that can support JSRV replication in vitro has limited the use of cell lines in OPA research. Primary OPA tumour cells or precision cut lung tumour slices can help overcome these issues [8,9], but extended in vitro culture typically leads to a reduction or cessation in virus production. Precision cut lung tumour slices also fail to replicate organ-level disease progression and do not enable monitoring of the disease in relation to clinical or field studies.

Immunodeficient [10] and immunocompetent [11] mouse models have also been employed in OPA research. Studies have shown that intra-nasal administration of adeno-associated virus vectors encoding the oncogenic JSRV Env protein leads to the formation of lung adenocarcinomas that are comparable to those found in sheep and humans [10,11]. However, the same limitations that caution over extrapolating the relevance of immunological phenomena between mouse models and man [12] are equally applicable to their use in the context of OPA research. Furthermore, the small size of mice presents practical limitations for their use in testing human anti-cancer therapeutics or novel techniques.

To address these challenges, researchers have developed in vivo ovine experimental JSRV infection models. Early studies demonstrated that the intra-tracheal injection of OPA tumour homogenates, cells, or supernatant from in vitro cultured OPA tumours into healthy lambs resulted in the formation of lung tumours [13,14]. Subsequently, it was found that OPA can be reproducibly induced by the inoculation of neonatal lambs with JSRV that was concentrated from lung fluid produced from OPA-affected animals [7,14,15,16,17]. Further refinement of the model was realised through the creation of oncogenic and infectious JSRV molecular clones, enabling virus production through the in vitro transfection of cell lines [18,19,20,21]; this meant that JSRV of a defined sequence and concentration could be used for in vivo studies. Studying the naturally occurring disease could potentially avoid the need for such experimental infection protocols. However, the identification of suitable cases for research presents a considerable challenge. Infected sheep rarely produce detectable circulating JSRV-specific antibodies [22,23,24], hampering the development of diagnostic serological blood tests. JSRV can be detected in blood samples using PCR [25,26], but low numbers of virally infected blood mononuclear cells contribute to a high level of false-negative results [27], rendering it unreliable for detecting subclinical disease [25]. The detection of JSRV in bronchoalveolar lavage samples offers increased test specificity [26]; however, sample collection requires sedation and may miss early cases as it only assesses a small portion of the lung. Imaging methods such as radiography and computed tomography (CT) have been suggested for use in OPA diagnosis but are costly, require specialised equipment, and need sedation and/or general anaesthesia. Trans-thoracic ultrasonography is a convenient imaging technique in screening for OPA; it can be performed rapidly on-farm with conscious animals and can detect OPA lesions as small as 2 cm in diameter involving the visceral pleura, although smaller tumours and those located more centrally or cranially may be undetectable [28]. Currently, post mortem examination is considered the gold standard technique for OPA diagnosis [29], with definitive confirmation obtained through the identification of JSRV in lung fluid or in tumours via immunoblotting [23], PCR [30], or immunohistochemistry (IHC) [3]. The inability to identify infected animals prior to significant lung tumour development is therefore a primary limitation of using field cases for experimental research. Sheep with overt naturally occurring OPA also often progress rapidly to clinical disease when put under stresses such as travelling. The presence or development of clinical signs poses animal welfare and ethical issues; natural cases are, therefore, not well-suited for in vivo studies.

A tractable model system should offer the facility to study OPA pathogenesis from the point of JSRV instillation to the development of gross OPA lesions and should minimise the potential for animals to develop clinical signs relating to respiratory insufficiency. Intra-tracheal instillation of JSRV has been the most commonly used JSRV infection route, which typically results in the formation of tumour foci throughout the lung fields. This is not the usual presentation of the natural disease and leads to the rapid onset of clinical signs, even when individual lesions are small, which prevents long-term study [3,14]. Previous models have also described bronchoscopic inoculation protocols and subsequent necropsy to follow early infection events (<10 days post-infection) in young lambs and adult sheep through immunohistochemical analysis [31]. Such approaches facilitate the exposure of defined local lung regions to high viral doses, maximising the chance of infection and potentially allowing for control over the site of tumour development.

Here, we report the development of an experimentally induced OPA model using bronchoscopic JSRV instillation into a defined segmental bronchus. Using three different viral instillation doses (low, intermediate and high), the initial characterisation of the model included calculating JSRV infection and OPA development rates between the groups and documenting the progression of OPA tumours through serial trans-thoracic ultrasound and CT imaging. Thoracic CT 3D reconstructions were generated to measure OPA tumour volumes and calculate tumour volume doubling times. We also compared ultrasound against CT in terms of its ability to accurately track tumour development over time and assess the level of agreement and association between the two imaging modalities. Importantly, no sheep in the study developed clinical signs of OPA. We believe that the range of OPA lesion types induced by this model replicates aspects of naturally occurring disease and will improve OPA research by providing novel insights into JSRV infectivity and OPA disease progression.

## 2. Materials and Methods

### 2.1. Animals

Lambs were obtained from the Moredun Research Institute’s high health status sheep flock and were Texel x Scotch Mule breed. They were vaccinated against Clostridia and Pasteurella and were weaned and housed together prior to transport to the University of Edinburgh’s Large Animal Research and Imaging Facility. Four-month-old lambs were arbitrarily separated into four age- and sex-matched (female and castrated male) groups, each containing 6 animals at the start of the experiment. The following groups were included in the study: PBS instillation (control group), JSRV low dose, JSRV intermediate dose, and JSRV high dose (treatment groups) (see Section 2.2 for viral doses). Throughout the study, sheep were housed in groups of at least 2 animals per pen, bedded on straw, with ad libitum access to food and water. Baseline CT scans (see Section 2.5) were used to screen sheep for pre-existing lung pathology, which would exclude them from the study. These CT scans were performed immediately prior to PBS or JSRV instillation. All control and JSRV-instilled sheep underwent the same bronchoscopic and imaging procedures throughout the 9-month experimental time period.

### 2.2. JSRV Production and In Vitro Quantification

JSRV was produced by transient transfection of 293T cells, as previously described [17]. Briefly, 293T cells (cultured in Iscove’s modified Dulbecco’s medium (Sigma, Gillingham, UK), supplemented with 4 mM of glutamine and 10% fetal bovine serum) were transfected with the JSRV molecular clone pCMV2JS_21_ using FuGene HD transfection reagent (Promega, Southampton, UK). The medium was replaced after 24 h and then harvested 48 and 72 h post-transfection and pooled. Harvested supernatant was clarified (300× *g*, 4 °C, 7 min), filtered through a 0.45 µm cellulose acetate filter (Sartorius, Gillingham, UK), and concentrated via ultracentrifugation at 40,000× *g* for 2 h. The viral pellet was subsequently resuspended in phosphate-buffered saline (PBS) at 100× concentration and stored at −80 °C.

As JSRV does not grow in cultured cells, TCID50 cannot be determined. Instead, the virus was quantified using a commercial reverse transcriptase (RT) assay (Roche colorimetric Reverse Transcriptase Assay, Merck, Gillingham, UK). The high, intermediate, and low viral doses were equivalent to 2000, 400, and 80 ng HIV RT, respectively, as assessed by the RT activity assay. The high dose is approximately equivalent to the dose given by intra-tracheal injection in previous studies [17].

### 2.3. General Anaesthesia

All imaging and bronchoscopic PBS or virus delivery procedures were conducted on anaesthetised animals. Concentrate feed was withheld the morning of anaesthesia. However, access to hay and water was permitted until pre-anaesthetic medication (medetomidine and ketamine) was administered via a pre-placed jugular venous cannula (Table 1). When sedation and recumbency were present (approximately 5 min), anaesthesia was induced with intravenous propofol, given until conditions for endotracheal intubation (with a cuffed tube) were present. Anaesthesia was maintained using end-tidal isoflurane concentrations of 1.5–2.0%. Isoflurane was vaporised in O_2_ or in an O_2_/air mixture and delivered using ‘circle’ breathing systems from anaesthesia workstations with integral ventilator (Aestiva/5, GE Datex-Ohmeda, Helsinki, Finland). Viral filters (Inter-Guard breathing filter, Intersurgical Ltd., Workingham, UK) were positioned between the endotracheal tube and breathing system. Volume-controlled positive pressure ventilation was implemented to achieve tidal volumes of 8–10 mL/kg and end-tidal CO_2_ tensions approximating 5.3 kPa. During anaesthesia, pulse rate, pulse oximetry, capnography, spirometry, and inspired and expired gases (O_2_, CO_2_ and inhalant anaesthetic agent) were monitored (S/5 Compact Anaesthesia Monitor, GE Datex-Ohmeda, Madison, WI, USA). Upon completion of all procedures, isoflurane was discontinued, and atipamezole was injected intra-muscularly. Ventilation was supported manually until spontaneous breathing of room air achieved SpO_2_ > 90%. The trachea was extubated once active chewing was present, and the sheep were monitored continuously thereafter until they could stand unassisted.

### 2.4. Trans-Thoracic Ultrasound Scanning

**Figure 1:**
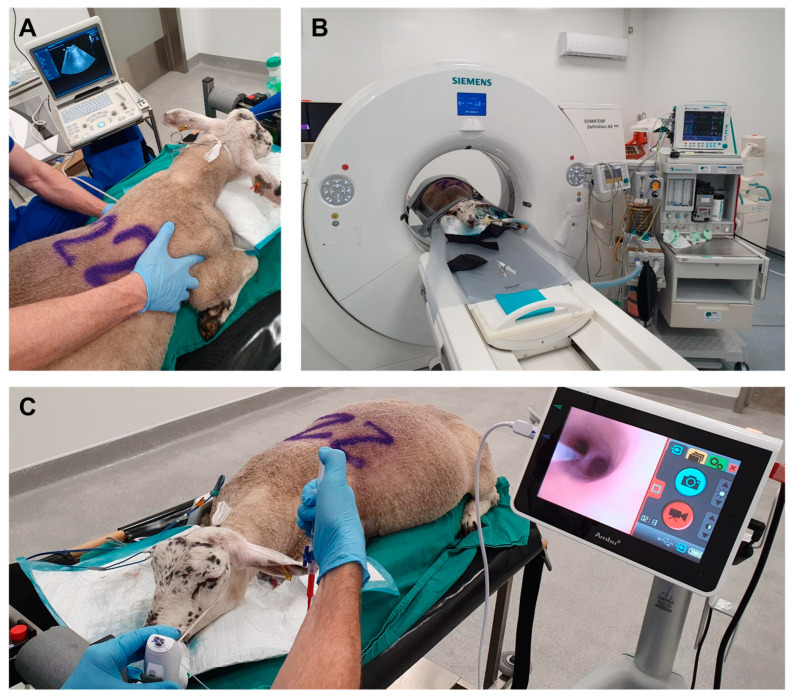
Pictures documenting sheep undergoing general anaesthesia for imaging and virus instillation. (**A**) Trans-thoracic ultrasound, (**B**) CT imaging, and (**C**) Bronchoscopy and virus instillation.

The thorax was clipped to facilitate ultrasound examination of the left and right lung fields. A 5.0–6.5 MHz microarray sector transducer connected to a real-time, B-mode ultrasound machine (Mindray DP-50, BCF Technology, Bellshill, UK) was used for all ultrasound procedures [32]. Ultrasound gel was applied to improve coupling between the probe and the skin. The ultrasound transducer head was positioned perpendicular to the skin surface, covering each intercostal space, and the lung fields were studied in both transverse and longitudinal planes through this acoustic window (Figure 1A). With the front leg extended forward, scanning started at the most accessible cranial intercostal space. Each intercostal space was sequentially scanned, moving caudally until the final intercostal space had been covered. The entire ultrasound examination was recorded as mp4 files using Elgato Video Capture software, version 1.15 (www.elgato.com, accessed on 16 August 2021).

Ultrasound scans were assessed for the presence of normal lung patterns and regions of consolidation. The ultrasound appearance of normal lung is characterised by the visceral pleural surface being identified as a hyperechoic line moving parallel to the thoracic wall during respiration. Fainter parallel hyperechoic lines (A lines) deep to the visceral pleural surface can be identified as reverberation artefacts caused by the ultrasound beam reflecting back and forth between the transducer head and the pleural surface. Immune cell infiltration and tumour cell proliferation, typical of OPA lesions, cause consolidation of the affected lung parenchyma. Typically, this consolidation produces a sharply defined hypoechoic area due to the transmission of sound waves through the tumour [28,33,34].

### 2.5. Computed Tomography Scanning

A multi-slice SOMATOM Definition AS 64-slice helical CT machine was used to obtain thoracic CT scans (Siemens Healthcare Ltd., Erlangen, Germany). Thoracic scans were performed using a fixed voltage of 120 kVp. Scans were performed with 3–5 mm collimation with 1 mm section thickness (Figure 1B). The window width and level were approximately 2000 and −500 HU, respectively, enabling simultaneous visualisation of normal lung, OPA lesions, blood vessels, bone, fat and muscle. All scans were recorded during an inspiratory breath hold with a target airway pressure of 20–30 cm H_2_O and were performed to include the entire thoracic cavity from the thoracic inlet to the last rib. Scans were assessed by a board-eligible veterinary radiologist who was blinded to the treatment groups.

CT scans were assessed for the presence of normal and abnormal lung patterns. The CT appearance of normal lung is characterised by lung parenchyma through which air-filled (black/hypoattenuating) bronchi and bronchioles run. On either side of the airways runs an associated artery and vein. Consolidated lung parenchyma, which occurs in OPA lesions resulting from tumour cell proliferation, causes these tissue areas to become hyperattenuating, with a similar density to that of the surrounding pulmonary vasculature. This loss of pulmonary vasculature definition, with preservation of airways, produces an alveolar lung pattern containing air bronchograms. Large, consolidated lesions may also cause small airways to collapse, leading to the loss of airway patterns. Hazy, ‘ground-glass’ hyperattenuating areas, with bronchial and vascular structures preserved, can also be detected typically at the periphery of consolidated lesions; these areas can represent the existence of diffuse regions of small neoplastic foci or pneumonia.

### 2.6. Bronchoscopy

Bronchoscopy was performed to instil PBS (control group) or JSRV (treatment groups) into the right cardiac lung lobe [35]. The bronchoscope (aScope 4 Broncho Slim 3.8/1.2, Ambu, Ballerup, Denmark) was introduced via the endotracheal tube and positioned within the segmental bronchus of the right cardiac lung lobe. A sterile instillation catheter (Ves Custom Optics, Welshpool, UK) was introduced down the working channel of the bronchoscope. Virus aliquots remained frozen on dry ice until immediately prior to use, when they were defrosted on wet ice. A total of 1 mL of either sterile PBS or virus was drawn up into 2.5 mL luer lock syringes and attached to the instillation catheter for delivery. The total 1 mL volume was delivered in approximately 200 μL bolus amounts to subsegmental bronchi leading from the segmental bronchus (Figure 1C). This volume was followed by a bolus of air to expel any remaining fluid from the catheter. Video recordings were captured as mp4 files.

### 2.7. Post Mortem Examination

All PBS- and virus-instilled sheep were euthanised when imaging results were consistent with a large localised OPA tumour or at a maximum time point of 9 months post-JSRV instillation. Euthanasia was performed using intravenous sodium pentobarbital (Pentoject; Animalcare, York, UK) at 80 mg/kg. The trachea, lungs, and caudal mediastinal lymph nodes were removed from the thoracic cavity. Post mortem evaluation of gross specimens was performed, which included photographic documentation of the lungs and any associated pathology (dorsal and ventral views were taken). Left and right cardiac lobes were inflated with 30% sucrose in PBS, mixed 2:1 with optimal cutting temperature tissue embedding matrix (CellPath, Newtown, UK), frozen on dry ice and stored at −80 °C. Eight fresh tissue samples were taken from the remaining lung lobes from specific pre-determined areas [17] (Supplementary Figure S1). Two further samples were taken from the caudal mediastinal lymph node. The fresh tissue samples were divided in two and either placed in 4% formaldehyde (Genta Medical, York, UK) for at least 24 h or snap frozen in liquid nitrogen. Later, samples from the frozen lobes were fixed and sectioned; at least 4 samples were taken from the right cardiac lobe (L3, the instilled lobe) and at least 1 sample was taken from the contralateral left cardiac lobe (L9). In total, at least 13 samples were prepared from defined locations of every lung.

### 2.8. Histopathology and Immunohistochemistry

Following fixation, tissue samples underwent routine processing through graded alcohols prior to embedding in paraffin wax. Four-micron tissue sections were cut and stained with haematoxylin and eosin (H&E). In addition, each sample was subject to IHC to label JSRV-positive cells and enable the detection of single or small groups of affected cells that can be missed on H&E stained sections [17].

For SU immuno-labelling, 4 µm tissue sections were deparaffinised and rehydrated. Antigens were retrieved using citric acid buffer (pH 6) and autoclaved for 10 min at 121 °C. Endogenous peroxide activity was inhibited by the addition of 3% hydrogen peroxide in methanol for 20 min. Slides were placed into a Sequenza rack (Thermo Scientific, Oxford, UK), washed twice with tris buffered saline (TBS), and then incubated with 0.1% Triton X-100 (diluted in TBS) to permeabilise the cells. After 30 min, non-specific labelling was blocked by the addition of BlockAid (Invitrogen, Paisley, UK) for 60 min at room temperature. Anti-SU primary antibody, a pool of two monoclonal antibodies (clones B3 and C9) that recognise the JSRV envelope SU protein (a kind gift from Sarah Wootton) [36], was diluted to 1:100 in BlockAid and added to the slides. Negative controls consisted of adding antibody diluent containing no primary antibodies and also the addition of primary antibody to lung samples known to be free of OPA. Positive controls consisted of the addition of primary antibody to confirmed natural OPA cases. Slides were incubated overnight at 4 °C. The slides were washed with TBS and then incubated with Dako EnVision^+^ System-HRP labelled polymer anti-mouse antibody (Dako, CA, USA) for 60 min at room temperature. The slides were washed in TBS and incubated with Dako chromophore/substrate solution (1:50) for 10 min. The slides were rinsed in water and then counterstained in hematoxylin, dehydrated, and mounted with coverslips using DXP mountant (Sigma-Aldrich, Glasgow, UK).

A board-certified veterinary pathologist, who was blinded to the treatment groups, examined the slides and reported on the histopathology and immunohistochemistry. Cases were diagnosed as OPA-positive based on the identification of JSRV-positive cells on IHC. OPA-positive sheep were then sub-classified into 3 categories based upon gross post mortem examination findings: (a) localised advanced lesions present in greater than half of the instilled lobe; (b) small early lesions with a width of less than 2 cm visible on the pleural surface of the instilled lobe; (c) no gross lesions.

### 2.9. CT Image Analysis

DICOM data were imported into Analyze 12.0 software (AnalyzeDirect, Overland Park, KS, USA) for performing quantitative analysis. Segmentation was performed by drawing separate regions of interest (ROI) comprising the trachea, mainstem bronchi, and normal lung parenchyma. No attempt was made to sub-segment individual lung lobes. Mainstem bronchi and smaller airways were included in the segmentation beyond the level of the carina and included those ventilating the right apical lung lobe. Other intra-thoracic structures, such as the heart, large blood vessels, and structures contained within the mediastinum, were excluded from analysis. All parenchymal tissue judged to be abnormal and consistent with an OPA lesion (consolidated tissue, tumour cells, and tumour microenvironment) was segmented into a separate ROI by assessing appearance, attenuation, and the presence of abnormal lung and vascular patterns.

**Figure 2:**
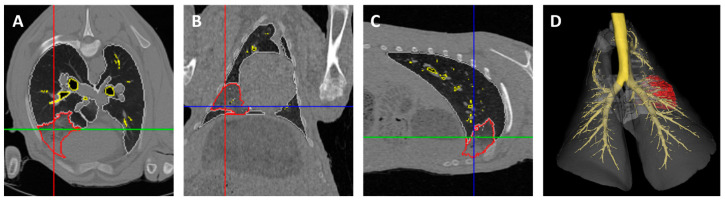
CT image analysis for the identification of regions of interest and tumour volumes. Each CT slice was segmented to create regions of interest, including the trachea, mainstem bronchi, small airways, left and right normal lungs, and tumour areas. Blue, green and red lines indicate CT slices corresponding to the shown axial (**A**), coronal (**B**) and sagittal (**C**) planes. (**D**) Final 3D reconstruction, dorsal view. Trachea, mainstem, and segmental and subsegmental bronchi (yellow); left, right, and accessory lung lobes (grey); right lung tumour (red).

Segmentation was performed on consecutive 2D CT image slices to obtain 3D volumes for each of the different ROIs. Segmentation was performed utilising views in all three imaging planes (axial, coronal, and sagittal). ROI delineation began by manually placing a ‘seed’ pixel on the structure of interest. Next, the intensity threshold was expanded to grow the seed boundary to encapsulate adjacent pixels until the structure boundary was defined. To restrict the seeded region from growing into neighbouring structures that shared similar pixel intensity values, manual limits were drawn between boundaries. ROIs were propagated to adjacent slices and adjusted to follow structure boundaries. A final visual assessment of ROIs in 2D and 3D allowed manual editing to refine ROI boundaries and finalise the 3D volumes (Figure 2).

### 2.10. Statistical Analysis and Calculation of Tumour Volumes

Comparison of sheep weight gain in each group was performed using a mixed-effects analysis followed by a Tukey’s multiple comparison test to compare all groups to each other at each time point (*p* values of ≤0.05 were deemed statistically significant). Tumour volume doubling times were calculated using the approximation formula developed by Schwartz [37]. Association and agreement between ultrasound and CT data, respectively, were assessed using Pearson correlation analysis to determine the linear relationship between each variable and a Bland–Altman analysis to visualise the relationship between measurement differences and averages [38].

## 3. Results

### 3.1. Local Bronchosopic Instillation Resulted in High Overall JSRV Infection and OPA Development Rates

Bronchoscopic JSRV instillation into the subsegmental bronchus of the right cardiac lung lobe was successful in all sheep, with no complications noted either during the procedure or following recovery from anaesthesia. Definitive OPA diagnosis was based on the presence of JSRV-infected cells detected by IHC using an antibody raised against the JSRV envelope SU protein. IHC was assessed by an experienced pathologist to confirm that the cell morphology and pattern of immunolabelling were consistent with tumour cells. The systematic sampling protocol from all lung lobes at post mortem examination facilitated the assessment of the distribution of JSRV-infected cells beyond the original area of virus instillation in the right cardiac lung lobe (Table 2). OPA tumour cells were cuboidal to columnar in nature, and immunolabelling was strongest along the plasma membrane, particularly at the apical surface, and variably present in the cytoplasm. In early tumours, the cancer cells were present as single cells or small clusters of up to approximately five cells, which were either attached to the alveolar wall or present within the alveolar space. More advanced tumours were characterised by larger clusters of cells within alveolar spaces or tumour cells arranged in single or multiple layers around a central area of fibrovascular stroma.

Whilst no JSRV-positive cells were identified in the control animals (sheep No. 1–6), in total, IHC diagnosed OPA in 16/18 (89%) of the JSRV-instilled animals. All three viral doses produced a range of OPA lesion types, including microscopic disease and gross tumours. However, larger lesions were more frequently identified in the low and intermediate viral dose groups. Of the 16 sheep diagnosed with OPA, 5 (31%, sheep No. 14, 17, 18, 20, and 24) developed advanced localised lesions consistent with OPA diagnosis in the instilled lobe; 1 of these sheep (sheep No. 14) also developed advanced lesions in the contralateral lobe. Two sheep (13%, sheep No. 23 and 25) had small early lesions consistent with OPA diagnosis, whereas nine (56%, sheep No. 13, 16, 21, 22, and 26–30) had no gross lesions (Table 2). Sheep 22 had two small lesions, each of a few mm in length, visible on the pleural surface of the instilled lobe, but was not classed as a gross lesion.

**Figure 3:**
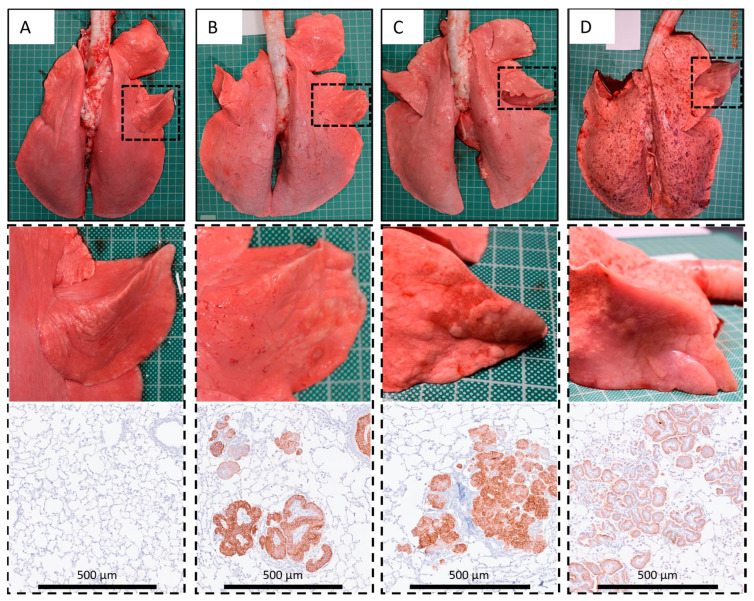
Gross post mortem images and representative IHC results. (**A**) Control (sheep No. 3). (**B**) OPA-positive: no gross lesions, JSRV positive cells detected by IHC (sheep No. 28). (**C**) OPA-positive: small early lesions with a width of <2 cm visible on the pleural surface, JSRV-positive cells detected by IHC (sheep No. 25). (**D**) OPA-positive: localised advanced lesions affecting greater than half of the affected lobe, JSRV-positive cells detected by IHC (sheep No. 20). IHC was performed using an antibody raised against the JSRV envelope SU protein. Pleural surface mottling apparent in the lungs in panel D represents post-euthanasia artefact.

Histological evidence of spread to other cranioventral lung lobes was apparent for all sheep with advanced lesions but was otherwise not a feature (i.e., any evidence of infection remained localised to the instilled lobe). The size of the microscopic tumour foci in other lobes ranged from a few cells labelled by IHC to obvious small lesions (Figure 3). All mediastinal lymph nodes examined in all animals were negative according to IHC for JSRV-infected cells (Supplementary Figure S2).

### 3.2. All Sheep Gained Weight and Remained Subclinical during the Study Period

**Figure 4:**
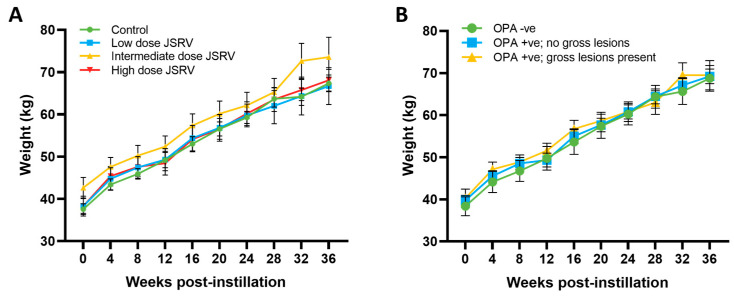
Sheep weight gain over the course of the study. (**A**) Average sheep weight gain with respect to treatment group. Mixed-effects analysis followed by a Tukey’s multiple comparison test, comparing all of the different groups to each other at each time point (mean ± SEM, n = 3–6). (**B**) Average weight gain with sheep separated into groups by tumour status at the time of euthanasia. Mixed-effects analysis followed by Tukey’s multiple comparison test, comparing each of the different groups to each other at each time point (mean ± SEM, n = 2–13).

JSRV-instilled animals have the potential to develop clinical signs of OPA, such as weight loss, increased respiratory rate and effort, cough and fluid produced by the lungs draining from the nostrils [29]. However, none of the animals exhibited clinical signs of disease during the study as the sheep with larger tumours were culled before they could progress to overt disease. Throughout the 9-month study period, all of the animals maintained a normal appetite and continued to gain weight, with no statistical difference identified between the controls or JSRV-instilled groups (Figure 4A). No statistical difference in weight gain was observed between those sheep that were classified as OPA-negative and OPA-positive (Figure 4B and Figure S3).

### 3.3. Ultrasound and CT Are Able to Identify Changes Consistent with OPA Diagnosis

All CT scans were assessed for the presence of normal and abnormal lung patterns. A preliminary CT-based diagnosis of OPA was given to sheep identified as having areas of hyperattenuating lung in the instilled right cardiac lung lobe. Typical advanced lesions appeared as large, consolidated regions causing small airways to collapse, leading to the loss of airway patterns. Ultrasound scans were assessed for the presence of normal lung patterns and regions of consolidation in the instilled lung lobe.

**Figure 5:**
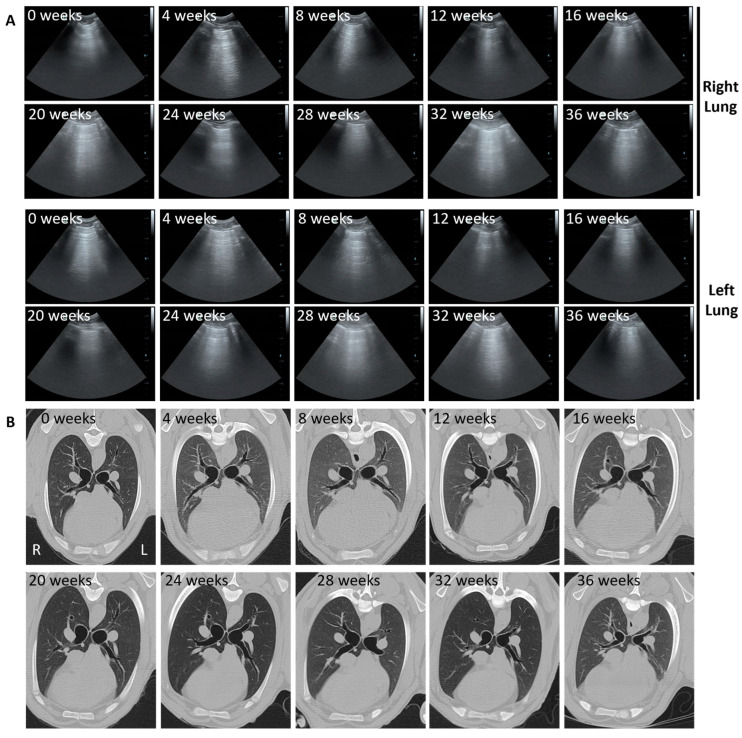
Normal thoracic ultrasound and CT appearance of ovine lungs from a control sheep over 9 months. (**A**) Transverse ultrasound images taken at the 4–6th intercostal spaces in the region of the cardiac lung lobes. An uninterrupted hyperechoic line consistent with the normal appearance of the visceral pleural surface can be seen at all time points. (**B**) Axial CT images highlighting the left and right cardiac lung lobes. Normal lung characterised by hypoattenuating lung parenchyma, through which air-filled (black) bronchi and bronchioles run, can be seen at all time points.

None of the sheep in the control group developed either ultrasound or CT imaging changes consistent with OPA development in the right cardiac lobe (Figure 5).

**Figure 6:**
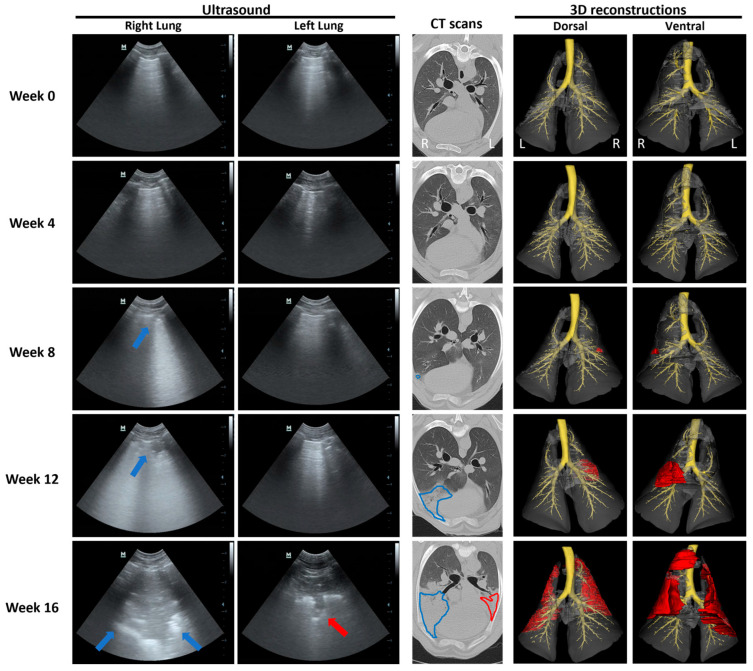
Serial ultrasound, CT and 3D reconstructions documenting OPA tumour development. Transverse ultrasound images were taken at the 4–6th right and left intercostal spaces in the region of the cardiac lung lobes. Blue arrows indicate progression of a right cardiac lobe OPA lesion from an irregular hyperechoic pleural line with B lines at 8 weeks to a large hypoechoic lesion at 16 weeks post-JSRV instillation. Red arrow indicates a left cardiac lobe OPA lesion identified at 16 weeks post-JSRV instillation. Axial CT images highlighting the left and right cardiac lung lobes. Blue and red lines highlight the progression of right and left OPA lesions, respectively. CT 3D reconstructions include the trachea, mainstem, segmental, and subsegmental bronchi (yellow); left, right, and accessory lung lobes (grey); left and right lung tumours (red).

**Figure 7:**
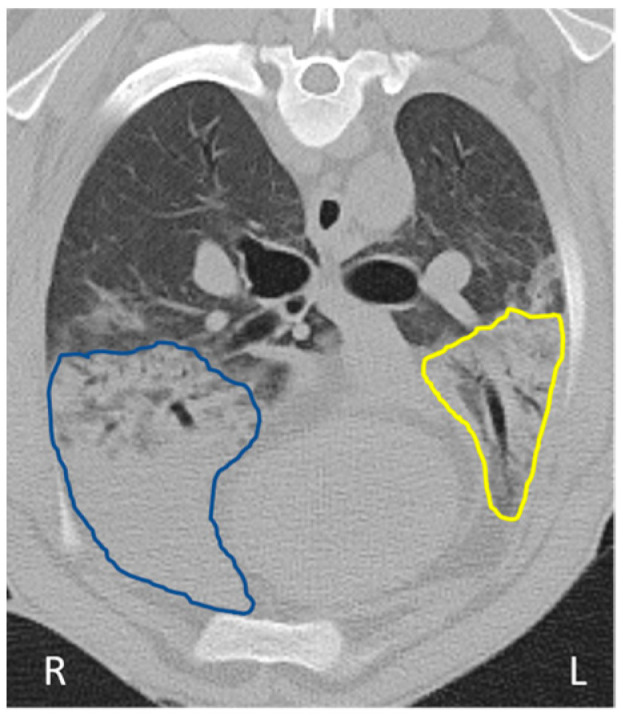
CT image documenting difficulties in CT-based OPA diagnosis. Axial CT image highlighting the left and right cardiac lung lobes from sheep 17. The region outlined in blue highlights hyperattenuating lung in the instilled right cardiac lung lobe, subsequently confirmed as OPA. The region outlined in yellow highlights hyperattenuating lung in the contralateral left cardiac lung lobe that was grossly normal and demonstrated no evidence of OPA by IHC.

Our results indicated that it was possible to track OPA tumour development over time in the instilled lobe using serial ultrasound and CT imaging (performed every 4 weeks post-instillation) in the seven cases that developed gross pathological OPA lesions (as confirmed at the post mortem examination). In all cases, consolidated right cardiac lung regions diagnosed on CT and ultrasound were confirmed as OPA-positive on IHC. Two of these sheep (sheep No. 14 and 17) also had CT changes consistent with OPA development in the contralateral left cardiac lung lobe (Figure 6 and Figure 7). While IHC diagnosed OPA in this lobe in sheep 14, sheep 17 was OPA negative. In the histological assessment of H&E stained sections, this sheep had small numbers of pulmonary alveolar macrophages and mild lymphocytosis; no ultrasound or gross post mortem changes were identified.

A representative example of OPA tumour development (sheep No. 14) is shown in Figure 6. Ultrasound images of the right lung fields at 8 weeks post-JSRV instillation show an irregular hyperechoic pleural surface with B lines. At 12 weeks, a small hypoechoic region extending less than 0.5 cm into the lung parenchyma with peri-lesional B lines was identified. By 16 weeks, this had developed into a large hypoechoic lesion extending approximately 4 cm into the lung parenchyma. Similar changes were evident in the left lung fields at 16 weeks. These changes were all identified in the ventral regions of the 4–6th intercostal spaces and would be consistent with the cardiac lung lobes. CT images show the progression of an area of hyperattenuation in the ventral portions of the right cardiac lung lobe from 8 weeks onwards. By 16 weeks, the area of hyperattenuation occupied the entire right cardiac lung lobe and is represented by an alveolar lung pattern with the presence of air bronchograms. Similar changes were also evident in the left cardiac lobe at 16 weeks (Figure 6). The CT images were used to generate thoracic CT 3D reconstructions, enabling 3D visualisation of tumour development over time (Figure 6 and Figure S4–S9).

### 3.4. OPA Tumour Growth Rates Are Variable but Can Be Rapid

**Figure 8:**
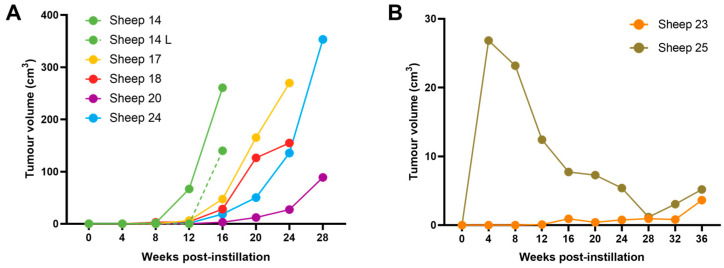
OPA tumour volumes. (**A**) Graph showing right cardiac lobe tumour volumes from all 5 sheep that were identified at post mortem examination as having advanced localised gross OPA lesions. The gross lesion that developed in the contralateral left cardiac lung lobe of sheep 14 is also shown (sheep 14 L, dashed line). (**B**) Graph showing tumour volumes from the 2 sheep that had gross post mortem lesions with <2 cm width visible on the pleural surface.

The tumour volume doubling time (based on the 3D reconstructions) for the five sheep that developed advanced localised right cardiac lung lobe OPA lesions (which were confirmed at post mortem examination) was 14.8 ± 2.1 days (Table 3). The mean right cardiac tumour volume was 225.5 ± 46.5 cm^3^ at the time of euthanasia (Figure 8A). Interestingly, even though JSRV was delivered locally to the right cardiac lung lobe, 1 out of these 5 sheep also developed gross lesions in the contralateral, left cardiac lung lobe. This lesion developed rapidly within the 4 weeks prior to euthanasia, at which point the tumour volume measured 139.9 cm^3^ (Figure 8A).

The earliest CT imaging changes consistent with OPA development were identified at 4 weeks post-instillation (sheep No. 25). At this time point, the presumptive tumour volume was 26.8 cm^3^. However, the volume subsequently decreased and, at the time of euthanasia (36 weeks post-instillation), was estimated from CT imaging as only 5.2 cm^3^. A further sheep (sheep No. 23) had a small area of hyperattenuation at the ventral portion of the right cardiac lobe that remained static until 32 weeks post-instillation, with an increase in size being identified at 36 weeks, which measured 3.64 cm^3^ (Figure 8B). Importantly, both sheep had small gross lesions consistent with OPA tumours at post mortem examination (<2 cm width visible on the pleural surface) located in positions consistent with the CT changes.

### 3.5. Ultrasound and CT Are Highly Correlated in Their Ability to Track Tumour Development over Time

Using ultrasound video recordings, the maximum depth (from the visceral pleural surface into lung parenchyma) of suspected hypoechoic OPA lesions in the regions of the right and left cardiac lung lobes (4, 5 and 6th intercostal spaces) was measured. Similarly, using axial CT images, the maximum depth (from the visceral pleural surface into lung parenchyma) that a suspected OPA lesion extended was measured.

**Figure 9:**
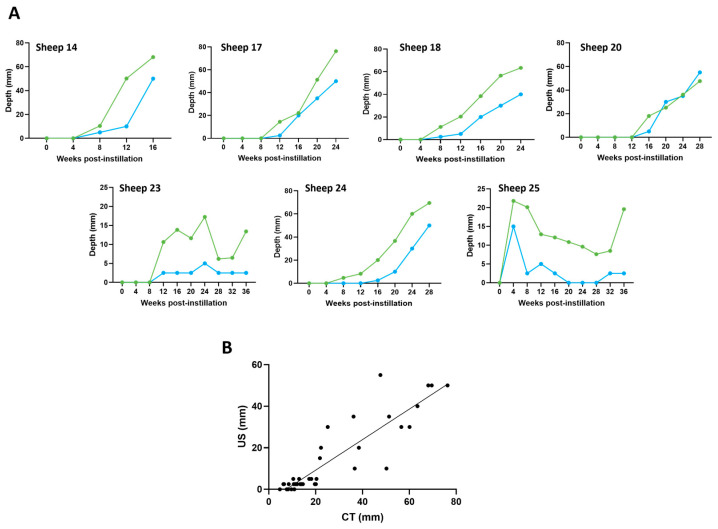
Changes in OPA lesion depth over time using CT and ultrasound. Measurements were taken from lesions identified within the right cardiac lung lobe. (**A**) Corresponding CT and ultrasound measurements were taken at each time point. CT tumour depth (green), ultrasound tumour depth (blue). (**B**) Pearson correlation scatterplot of paired CT and ultrasound measurements (R^2^ = 0.799; *p* < 0.0001).

The results indicated a strong significant association between paired CT and ultrasound maximum measurements at each time point (R^2^ = 0.799; *p* < 0.0001). The Bland–Altman plot indicated that 95% of the data points lay within two standard deviations of the mean difference. CT measurements were, on average, approximately 12.5 mm larger than the ultrasound measurements; in some sheep, there was strong agreement between these respective measurements, whilst in others, there was less agreement. In all but 5 out of 68 time points (sheep No. 24; right cardiac lobe at 8 and 12 weeks, sheep No. 25; right cardiac lobe at 20, 24, and 28 weeks), a lesion identified on CT was also detected on ultrasound (Figure 9).

## 4. Discussion

Here, we report the development of an experimentally induced OPA model that can be used to accurately monitor tumour development over an extended time period, which allows for samples to be taken from defined stages of disease progression. It is the first study to compare CT and ultrasound data to monitor OPA disease progression and lesion size. This model has the potential to generate new knowledge about OPA and contribute towards advancing research into the control of the disease.

In this study, we used bronchoscopic instillation of JSRV into a predefined lung region to induce OPA development in a specific lung lobe. To achieve this, we used small viral instillation volumes to facilitate precise targeting of subsegmental bronchi and reduce the risk of fluid spillover into neighbouring lung regions. We chose a defined subsegmental bronchus of the right cardiac lobe for JSRV instillation, as this lobe is commonly affected in naturally occurring cases. Due to its natural ventral dependence, it is also more likely to locally retain instilled fluid volumes. Although typically requiring general anaesthesia, bronchoscopy is a minimally invasive procedure that can be performed even in young animals. This localised delivery method holds advantages over previously described intra-tracheal JSRV instillation techniques, as the latter delivery method results in the formation of tumour foci throughout the lung fields, a presentation which does not typify the natural disease and can lead to a more rapid onset of clinical signs [14]. Bronchoscopic administration of JSRV has been reported previously for a study to investigate the effects of generalised lung injury (3-Methylindole administration) on JSRV infection rates [31]. In that study, researchers instilled JSRV into the accessory bronchus of the right apical lung lobe but did not use advanced imaging techniques to document lung changes and euthanised the animals 10 days post-infection before the development of OPA tumours.

We aimed to develop a model to replicate aspects of naturally occurring OPA, including factors such as variable and low incidence rates of gross tumour development, the presence of sheep with only histological evidence of disease and sheep with no evidence of disease [39]. In our study, OPA was identified in 16/18 (89%) of the JSRV-instilled animals. In previously reported JSRV infection models, OPA has been identified in 85–100% of animals following the intra-tracheal delivery of JSRV to neonatal lambs; however, OPA development rates were lower when older animals were used [16,40]. We chose to use 4-month-old lambs to ensure the bronchial tree was large enough to allow bronchoscopic localised JSRV delivery. Considering this technique proved successful in all sheep, in future studies, it will be interesting to perform the same procedure in younger animals to investigate what effect age has on JSRV infection and/or OPA development rates. Of the sheep diagnosed as OPA positive by 9 months post-instillation, seven had grossly visible lesions consistent with OPA diagnosis on post mortem examination, whereas nine had lesions found by IHC but no gross lesions. Given a longer study period, we might speculate that these animals would also have developed gross OPA lesions. Notably, we cannot rule out that the two instilled animals identified as OPA-negative may have had microscopic lesions that were missed by our sampling protocol. As is usual for natural cases, we did not detect mediastinal lymph node involvement in any of the positive cases [29]. The range of lesion types generated within this model provides an opportunity to study OPA disease progression, from JSRV infection right through to the development of microscopic tumour foci and gross lesions.

Our study showed that the development of OPA was variable between animals but did not suggest a direct relationship between the dose of instilled JSRV and tumour formation or their growth rate. Here, we chose a dose of virus (high dose group) similar to that which we had administered previously for intra-tracheal instillation experiments [17]; however, because instillation into a discrete region of a single lobe might result in a more rapid onset of tumour formation and clinical signs, we included two additional groups of animals instilled with more dilute virus doses at 20% (intermediate dose group) and 4% (low dose group) of the highest dose concentration. We anticipated that the inoculum containing the largest amount of virus would yield more sheep with tumours and also larger tumours than the lower amount of virus. Although JSRV infection rates were comparable between groups, we found that the highest viral dose did not produce larger or greater numbers of gross tumours. If an inverse correlation between viral dose and gross tumour formation were proven in future studies, one hypothesis to explain this might be that higher viral doses could induce innate or acquired immune responses, perhaps shifting the balance of cells in the local microenvironment towards inhibiting tumour formation. Besides the amount of instilled virus, other technical and biological/host factors may influence the likelihood of JSRV infection and/or the rate of OPA developing. To reduce variation between groups, the bronchoscopic instillation technique was standardised and conducted by a single veterinary surgeon experienced in bronchoscopy. Although JSRV instillations were conducted over 2 consecutive days, the viral doses used were all generated from a single pooled viral master stock. To reduce biological variability, all sheep were the same age and from the same flock. However, other biological/host factors are challenging to control for. These factors may include a variation in the proportion of susceptible target cells (proliferating type II pneumocytes or club cells) in the lungs of different sheep. Other host determinants, such as currently unidentified genetic factors, may also influence the susceptibility of sheep to JSRV infection and/or OPA tumour progression. A further potential factor is that the local microbiome may shape the dynamics of viral infection and OPA progression. It is recognised that airway bacterial community profiles in healthy sheep are highly variable over time, between individuals and spatially across lung segments [41]; the differential outcome of infection rates in our OPA model may reflect such differences. Adding credence to this suggestion is the observation that concurrent bacterial lung inflammation and damage are common features associated with naturally occurring OPA, and indeed, the spatial disposition of OPA and bacterial lung disease pathology caused by Pasteurella multocida and Mannheimia haemolytica share a cranioventral distribution.

Using IHC with an antibody that labels JSRV-positive cells, we investigated whether JSRV could be detected beyond the instilled right cardiac lung lobe. In the five sheep (sheep No. 14, 17, 18, 20, and 24), which developed large gross lesions in the instilled lobe, JSRV was also detected in other cranioventral lung lobes. Only one of these (sheep No. 14) developed a gross, cranioventral lesion outside of the instilled lobe. This mimics the presentation of natural cases in which cranioventral lobes are commonly affected. Previous studies have speculated that a gravitational influence on inhaled bioaerosols may contribute to specific lung regions being predisposed to the development of pathology [42]. However, as the bronchus leading to the accessory lung lobe is closest to the right cardiac instillation site, one would expect this lobe to be a common site for gross OPA lesion development in our study, which was not the case. No tumour spread was identified in the sheep identified as having either early lesions or no gross lesions. However, it should be noted that the number and extent of very small lesions would have been underestimated because only a small proportion of each lung lobe was sampled (typically 1 cm × 2 cm × 4 µm). Previous studies have demonstrated that OPA tumours are oligoclonal, supporting tumour expansion through new infection events rather than by metastatic spread [43]; therefore, the development of widespread tumour foci beyond the initial site of instillation likely developed through new infection and transformation events, i.e., resulting from the production of new viral particles by the initial JSRV-transformed tumour cells. However, additional studies would be necessary to confirm this.

An important ethical advantage of our protocol is that it allows studies of OPA progression without requiring the serial sacrifice of animals or the need for the disease to progress to a stage that results in clinical signs. The use of CT and ultrasound facilitates objective monitoring of tumour progression and enables informed decisions on when to euthanise animals before the development of clinical signs. CT was selected as the imaging modality to monitor tumour progression as it provided a comprehensive description of the extent of the OPA lesions occurring within the entire thorax. CT images also allowed the generation of 3D reconstructions in order to measure tumour volumes and calculate tumour growth rates over time; this is the first time that OPA lesion 3D reconstructions have been performed. OPA has been considered to be a slowly progressive disease of older animals until recent evidence from serial ultrasound scanning showed rapid, but variable, progression in some sheep [44]. The data presented here support this observation and indeed demonstrates that, in some instances, tumour volumes can double in under 2 days.

In all cases, consolidated right cardiac lung regions diagnosed on CT and ultrasound were confirmed as OPA positive on IHC. However, in one case (sheep No. 17) where CT had identified pulmonary changes suggestive of OPA in the contralateral left cardiac lobe, this was later confirmed as OPA-negative on IHC. This highlights the potential difficulty in obtaining a pre-clinical, imaging-based OPA diagnosis when assessing lung regions outside of the instilled lung region. The observed regression of CT and ultrasound imaging changes consistent with OPA lesions in one animal in this study is of particular interest and is consistent with previous studies showing evidence of OPA tumour regression [24,44]. Following euthanasia at 9 months post-instillation, using IHC, we were able to diagnose this case (sheep No. 25) as OPA-positive in the region of JSRV instillation, the same area where the regressing lesion was noted by serial CT imaging. However, unequivocal evidence, through obtaining biopsies to confirm the diagnosis at an earlier stage, is needed to verify that the CT and ultrasound changes observed were genuinely indicative of OPA tumour presence, growth, and subsequent regression.

This is the first study to compare serial CT and ultrasound data to monitor OPA disease progression and lesion size. CT has previously been used for monitoring disease development and progression in both experimentally infected JSRV animals [24] and in sheep born into an endemic JSRV infected flock [45]. However, CT is not suitable for on-farm disease surveillance, screening, or as a diagnostic test as it is cost-prohibitive and impractical for use as a field test [32]. Although trans-thoracic ultrasound can only detect lesions affecting the visceral pleural surface, it has proved relatively sensitive, rapid, and affordable for the on-farm screening of OPA [46]. Furthermore, results are obtained quickly and affordably from conscious sheep, enabling the screening of large flocks [33,47,48]. Ultrasound examination demonstrates good specificity for OPA diagnosis and can usually differentiate OPA from other common ovine lung diseases [46]. We compared the ultrasound identification of lesions within the right cardiac lobe to those lesions identified on CT. We demonstrated that small pleural irregularities (multiple B lines or irregular pleural surface) were identified in the right cardiac lobe by ultrasonography at the same stage that the earliest changes on CT were identified. Although it is not possible to definitively state the nature of the underlying pathology, with serial imaging, we showed that it is possible to track changes in these lesions over time.

Some variability between CT and ultrasound agreement was identified between different sheep and, in some instances, even within sheep at different time points. Possible reasons for this variation could include CT being conducted under breath-hold conditions compared to ultrasound being performed in ventilated animals, and/or inter- and intra-animal (longitudinal) variation in the mechanical properties of the lungs and tumour tissue, as well as small variations in the angulation of the ultrasound transducer probe. Indeed, intrinsic limitations in the ability of ultrasound to visualise deeper lung structures might also be a factor in the consistently lower estimates of tumour ‘size’ using this technique. Our method of trans-thoracic ultrasound offers ideal standardised conditions in that spontaneous movement is controlled, the fleece is clipped, and ultrasound gel is applied. This is more time-consuming and involved than the typical approach employed for screening flocks on-farm. Such preparation, and the use of a single experienced veterinary ultrasonographer throughout the study, likely contributed to our ability to demonstrate close association and agreement between these two imaging methods. Characterising the tissue pathobiology underlying particular ultrasound features remains an important and unmet need that could be addressed using this model system, which enables the gross and histological morphology of the lungs to be compared and correlated with recorded ultrasound images obtained in vivo. New imaging technologies, such as quantitative ultrasound [49], could be tested with this model to potentially improve upon current grayscale (B-mode) imaging.

Our described model represents a significant step forward in the development of a tractable system to study OPA pathogenesis. However, all models have potential limitations, and their use has to be considered in relation to the research question being investigated. Our model resulted in 5/18 of the JSRV-instilled sheep developing advanced localised lung tumours. This rate may not be considered appropriate for applications requiring the generation of gross tumours. Using younger animals and extending the period of observation post-infection might increase JSRV infection and OPA development rates. However, the discovery of fundamental drivers of susceptibility, including highly susceptible genotypes, would be key to developing the most appropriate and efficient experimental infection protocol. Other enhancements to the model could include varying the dose, volume, or frequency of JSRV delivery or adopting different delivery methods, such as trans-bronchial injections.

Through initiating tumour development within a particular lung lobe and monitoring animals from the point of JSRV instillation until the development of large localised primary and secondary lesions, this OPA model opens up new areas of research. Such investigations could potentially ascertain the molecular basis of disease susceptibility, lead to the discovery of biomarkers, or be applied as a challenge model for the development of vaccines. Serial respiratory sample collection, such as bronchoalveolar lavage fluid, exhaled breath, and nasal swabs, could be readily applied to our model. These types of samples could be used to track JSRV excretion and evaluate immune cell profiles or the expression of inflammatory mediators during disease progression. A major factor which has limited the control of OPA is the lack of a blood-based pre-clinical diagnostic test; the identification of novel blood-based diagnostic markers could resolve this issue. A key advantage of our OPA model is that serial blood samples can be obtained from before JSRV instillation right through to the development of large localised tumours. These unique samples are ideally suited for the identification of blood-based OPA diagnostic and/or disease progression biomarkers.

## 5. Conclusions

We report the development of an experimentally induced OPA model using bronchoscopic JSRV instillation into the segmental bronchus of the right cardiac lung lobe. The model replicates aspects of naturally occurring OPA, exhibiting high JSRV infection rates and generating different OPA lesion types, ranging from microscopic disease to gross tumours. It is the first study to describe serial ultrasound, CT imaging, and 3D reconstructions from the point of JSRV instillation to the development of advanced localised OPA tumours. We have shown that OPA tumour development can be accurately tracked using either CT or trans-thoracic ultrasound and that all of the JSRV-infected animals remained healthy, with no sheep developing clinical signs consistent with progressive OPA. We believe that this model can be used to significantly improve OPA research by providing novel insights into JSRV infectivity, OPA disease progression and its pathogenesis.

## Figures and Tables

**Table 1:** Techniques used to provide ovine anaesthesia and analgesia. * Until conditions for endotracheal intubation were present (i.m., intramuscular; i.v., intravenous).

Phase	Drug	Manufacturer	Dose (mg/kg)	Route
Sedation	Medetomidine	‘Medetor’; Virbac Ltd., Suffolk, UK	0.005	i.v.
	in combination with			
	Ketamine	‘Ketamidor’; Chanelle Vet Ltd., Hungerford, UK	0.5	i.v.
Induction	Propofol	‘Propofol’; Fresenius Kabi, Cheshire, UK	To effect * (e.g., 2–5)	i.v.
Maintenance	Isoflurane	‘Isofane’; Piramal Critical Care Ltd., West Drayton, UK		Inhaled
Recovery	Atipamezole	‘Atipam’; Dechra, Eurovet Animal Health BV, Bladel, The Netherlands	0.025	i.m.

**Table 2:** Final OPA diagnosis. Definitive OPA diagnosis was based on the identification of tumour cells and the detection of JSRV envelope SU protein using IHC. Within each treatment group, sheep are ordered on the basis of OPA status. Months post-instil indicates the time of euthanasia. OPA-positive sheep are sub-classified into 3 categories based upon a combination of the gross pathology findings and IHC results, namely, ‘Advanced lesions’ affecting greater than half of the instilled lobe, ‘Early lesions’ with a width of <2 cm visible on the pleural surface of the instilled lobe, and ‘No gross lesions’. In addition, the spread of JSRV beyond the instilled right cardiac lobe (L3) is indicated by the IHC results for each of the 10 lung samples. L1-L10 samples were collected according to the diagram in Supplementary Figure S1. No JSRV-positive cells (-/blue), 1–5 tumour foci per tissue section (+/tan), 5 or more tumour foci per tissue section (++/orange), not sampled (ns).

Group		JSRV Low Dose						JSRV Intermediate Dose						JSRV High Dose						Control
Sheep No.		14	18	17	13	16	15	24	20	23	21	22	19	25	26	27	28	29	30	1–6
Sex		M	F	F	M	F	M	M	F	M	F	M	F	M	M	M	F	F	F	M&F
Months post-instil		4	6	6	9	9	9	7	7	9	9	9	9	9	9	9	9	9	9	9
IHC Sample Site	L1	++	++	+	-	-	-	++	-	-	-	-	-	-	-	-	-	-	-	-
	L2	++	++	-	-	-	-	++	-	-	-	-	-	-	-	-	-	-	-	-
	L3	++	++	++	+	+	-	++	++	+	++	+	-	++	+	+	+	+	+	-
	L4	++	++	-	-	-	-	++	-	-	-	-	-	-	-	-	-	-	-	-
	L5	ns	-	-	-	-	-	-	++	-	-	-	-	-	-	-	-	-	-	-
	L6	ns	-	-	-	-	-	-	-	-	-	-	-	-	-	-	-	-	-	-
	L7	ns	-	-	-	-	-	-	-	-	-	-	-	-	-	-	-	-	-	-
	L8	ns	-	-	-	-	-	++	-	-	-	-	-	-	-	-	-	-	-	-
	L9	+	++	-	-	-	-	-	-	-	-	-	-	-	-	-	-	-	-	-
	L10	-	++	-	-	-	-	-	-	-	-	-	-	-	-	-	-	-	-	-
		**OPA Status**																		
Advanced lesions		✓	✓	✓				✓	✓											
Early lesions										✓				✓						
No gross lesions					✓	✓					✓	✓			✓	✓	✓	✓	✓	
Negative							✓						✓							✓

**Table 3:** Tumour volume doubling times for sheep that developed advanced localised OPA lesions within the right cardiac lung lobe.

Sheep No.	Tumour Volume Doubling Time (Days)
14	7.39
17	15.37
18	19.56
20	17.20
24	14.59

## Data Availability

Data are contained within the article or Supplementary Materials.

## Supplementary Materials

The following supporting information can be downloaded at: https://www.mdpi.com/article/10.3390/genes15081019/s1, Figure S1: Diagram of ovine lungs depicting sampling areas for histopathology; Figure S2: Representative mediastinal lymph node IHC. Figure S3: Weight gain for each individual sheep in the study; Figure S4: The 3D reconstructions from serial CT scans documenting OPA tumour development in sheep 17; Figure S5: The 3D reconstructions from serial CT scans documenting OPA tumour development in sheep 18; Figure S6: The 3D reconstructions from serial CT scans documenting OPA tumour development in sheep 20; Figure S7: The 3D reconstructions from serial CT scans documenting OPA tumour development in sheep 23; Figure S8: The 3D reconstructions from serial CT scans documenting OPA tumour development in sheep 24; Figure S9: The 3D reconstructions from serial CT scans documenting OPA tumour development in sheep 25.
