# Supplementary figures and images for: Tracking Ovine Pulmonary Adenocarcinoma Development Using an Experimental Jaagsiekte Sheep Retrovirus Infection Model
Source: Genes (Basel). 2024 Aug 2;15(8):1019. doi: 10.3390/genes15081019 (PMC11353984; doi:10.3390/genes15081019)

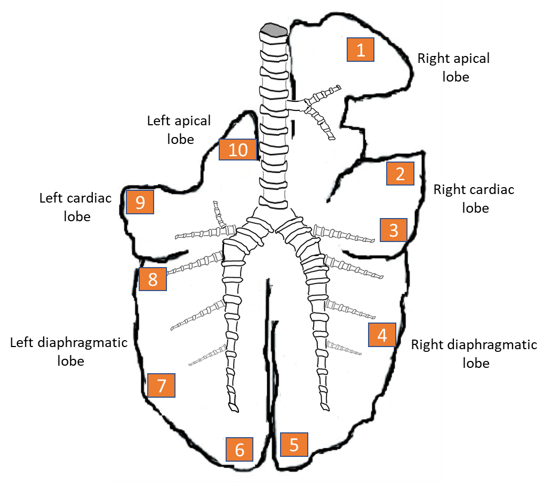

Supplement: Supplementary file 1 [file genes-15-01019-s001.zip › Supplementary figure S1.tif]

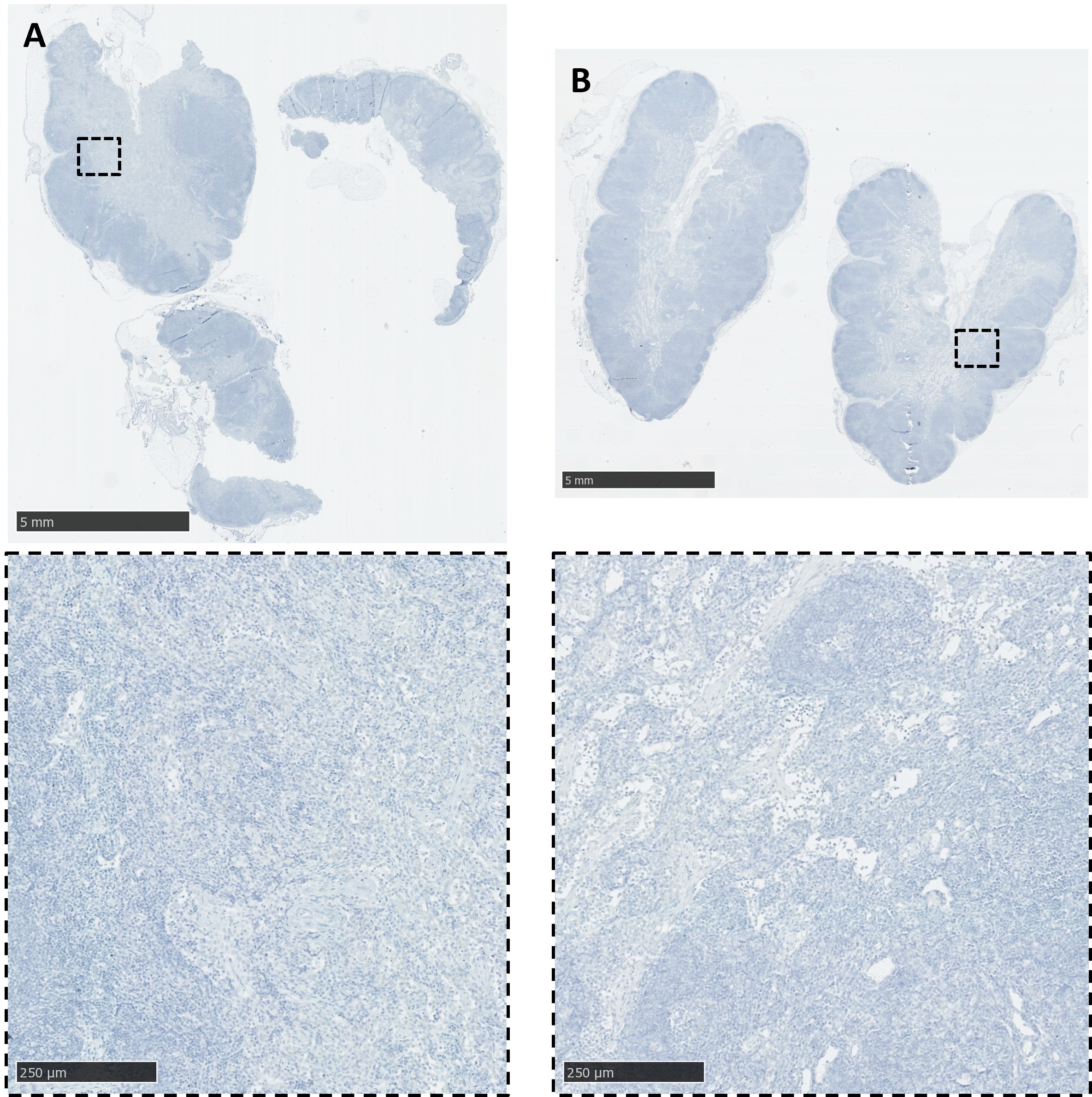

Supplement: Supplementary file 1 [file genes-15-01019-s001.zip › Supplementary Figure S2.tif]

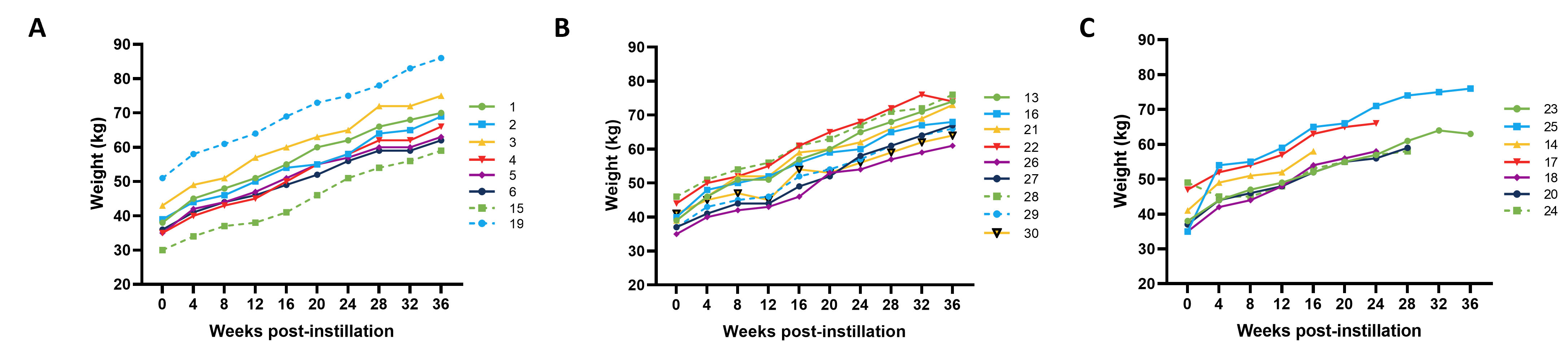

Supplement: Supplementary file 1 [file genes-15-01019-s001.zip › Supplementary Figure S3.tif]

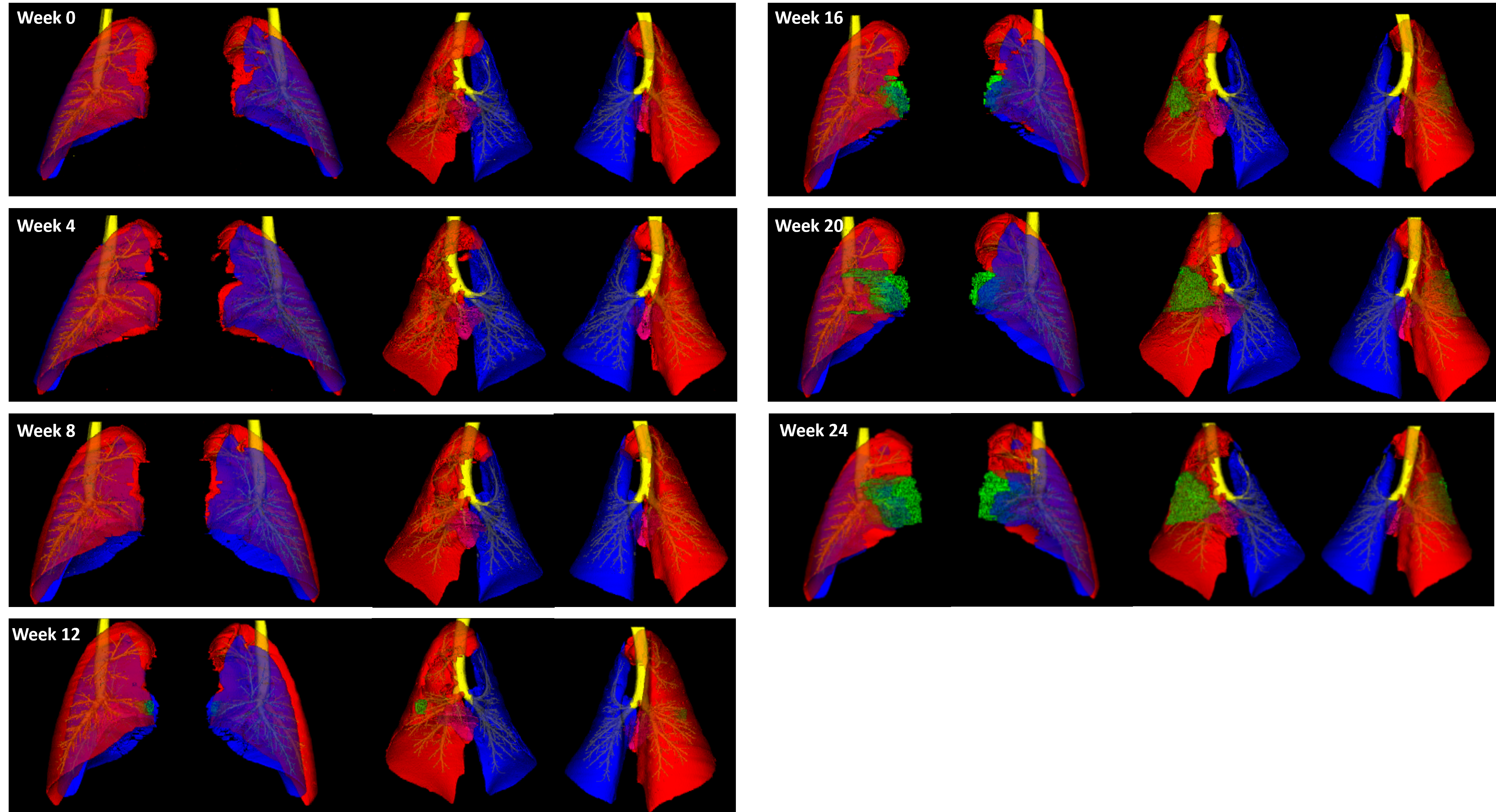

Supplement: Supplementary file 1 [file genes-15-01019-s001.zip › Supplementary Figure S4.tif]

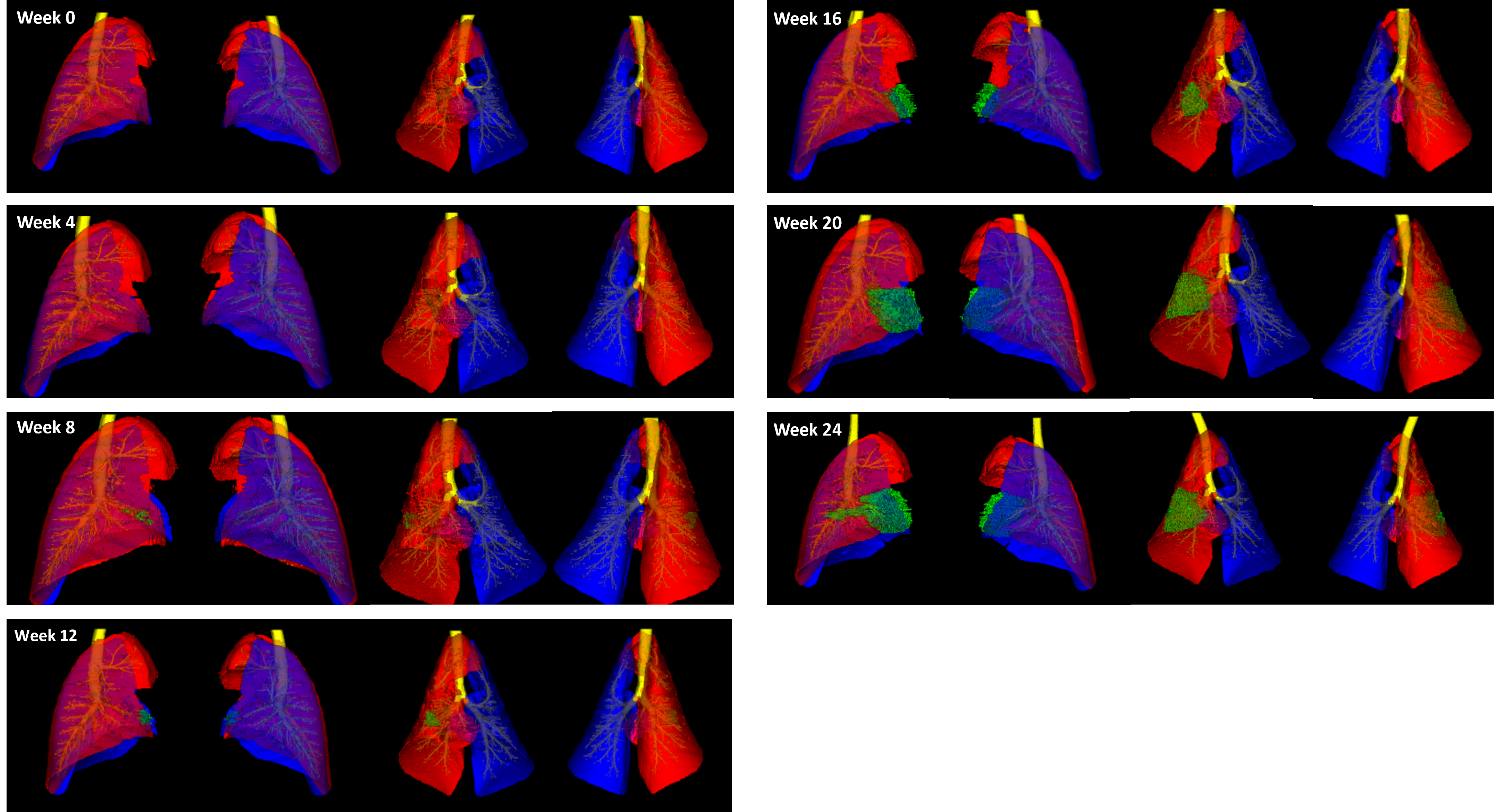

Supplement: Supplementary file 1 [file genes-15-01019-s001.zip › Supplementary Figure S5.tif]

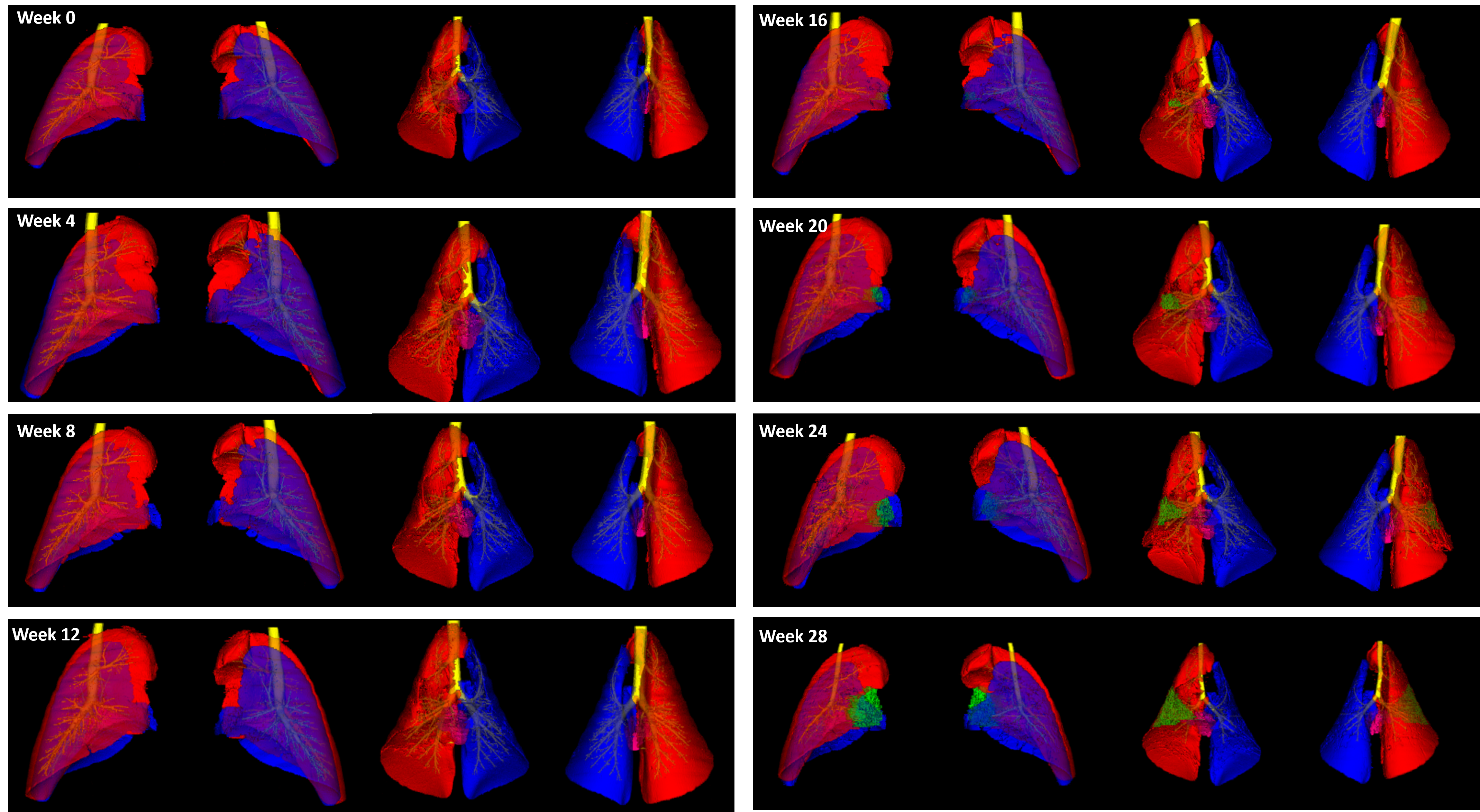

Supplement: Supplementary file 1 [file genes-15-01019-s001.zip › Supplementary Figure S6.tif]

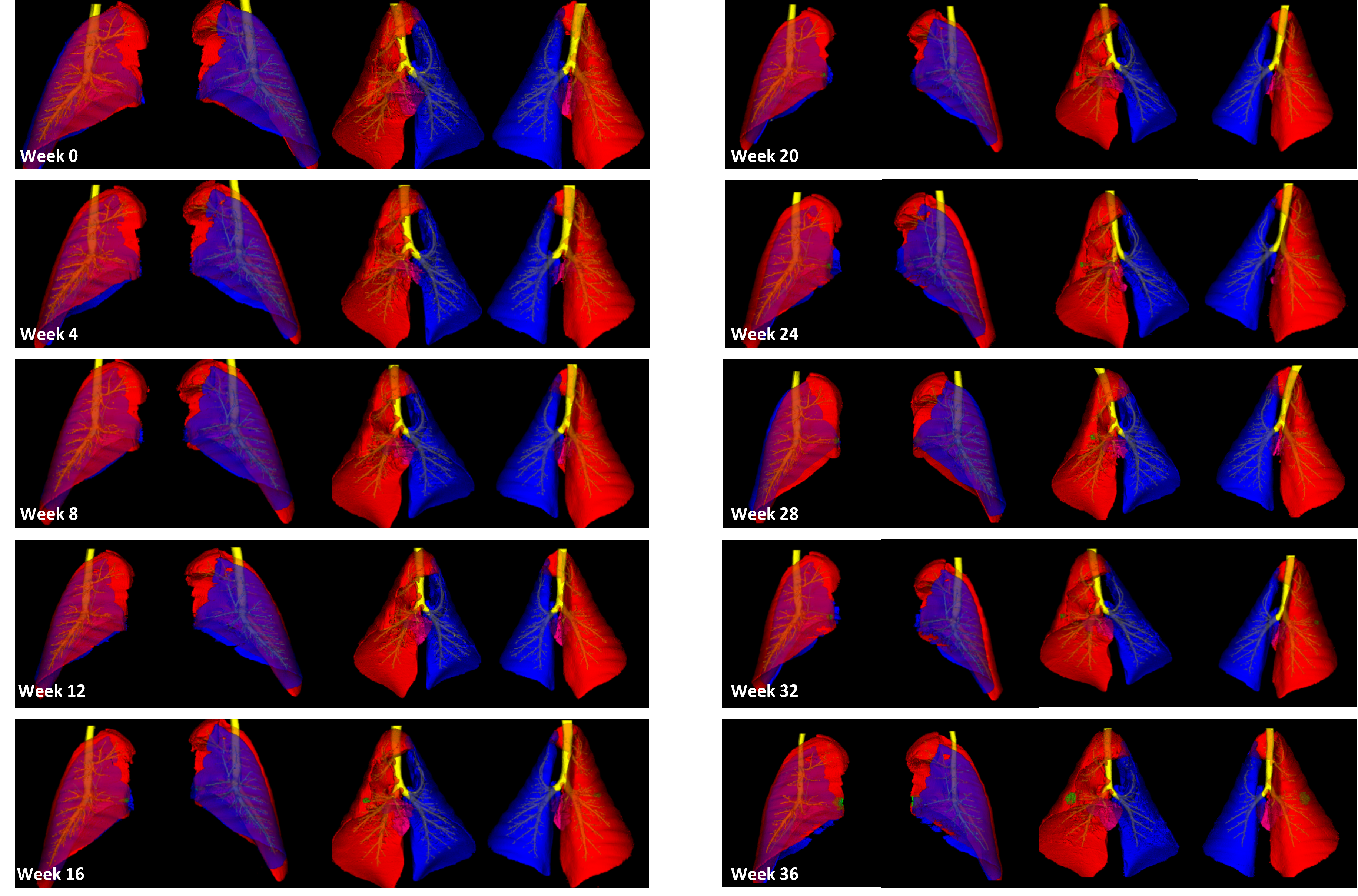

Supplement: Supplementary file 1 [file genes-15-01019-s001.zip › Supplementary Figure S7.tif]

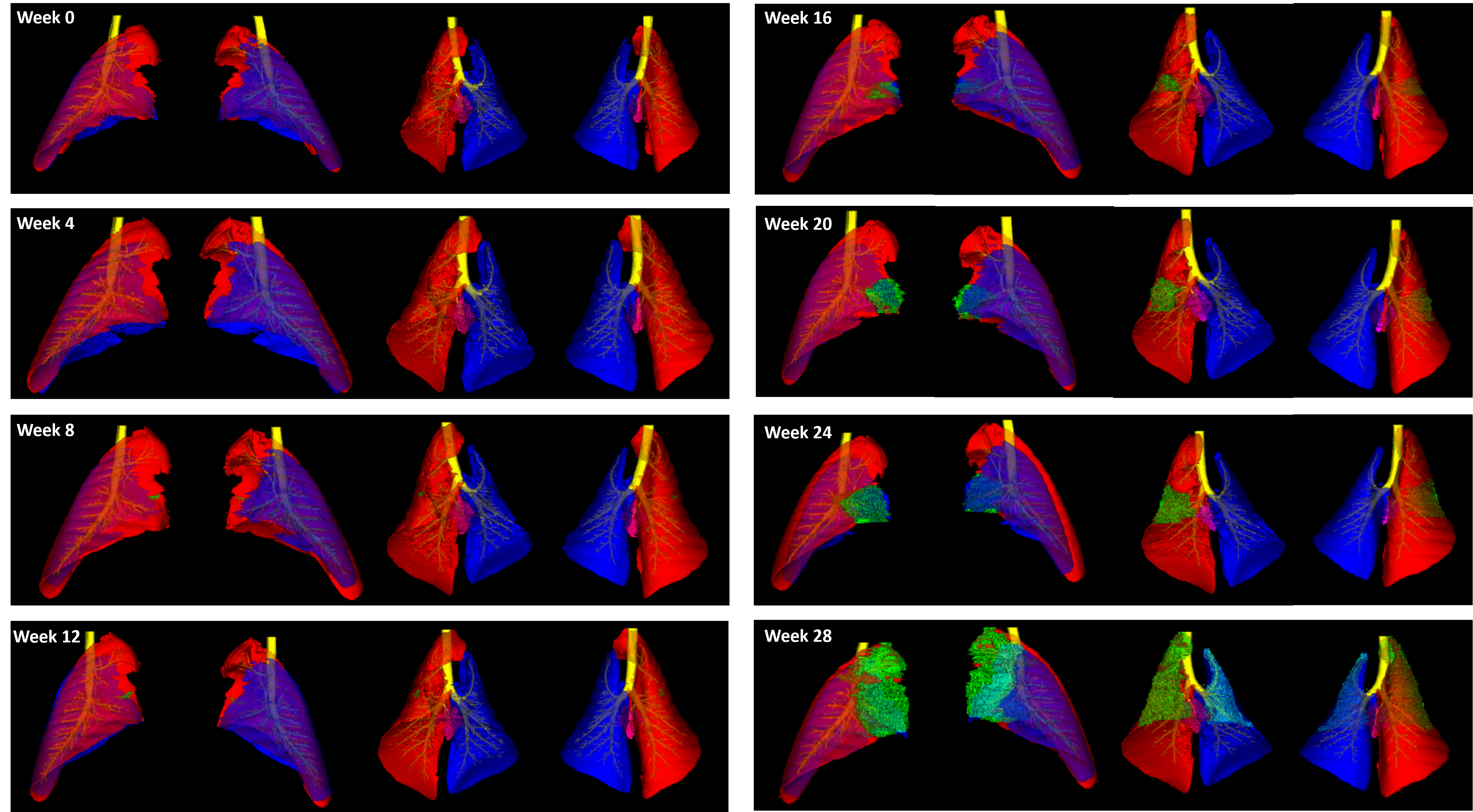

Supplement: Supplementary file 1 [file genes-15-01019-s001.zip › Supplementary Figure S8.tif]

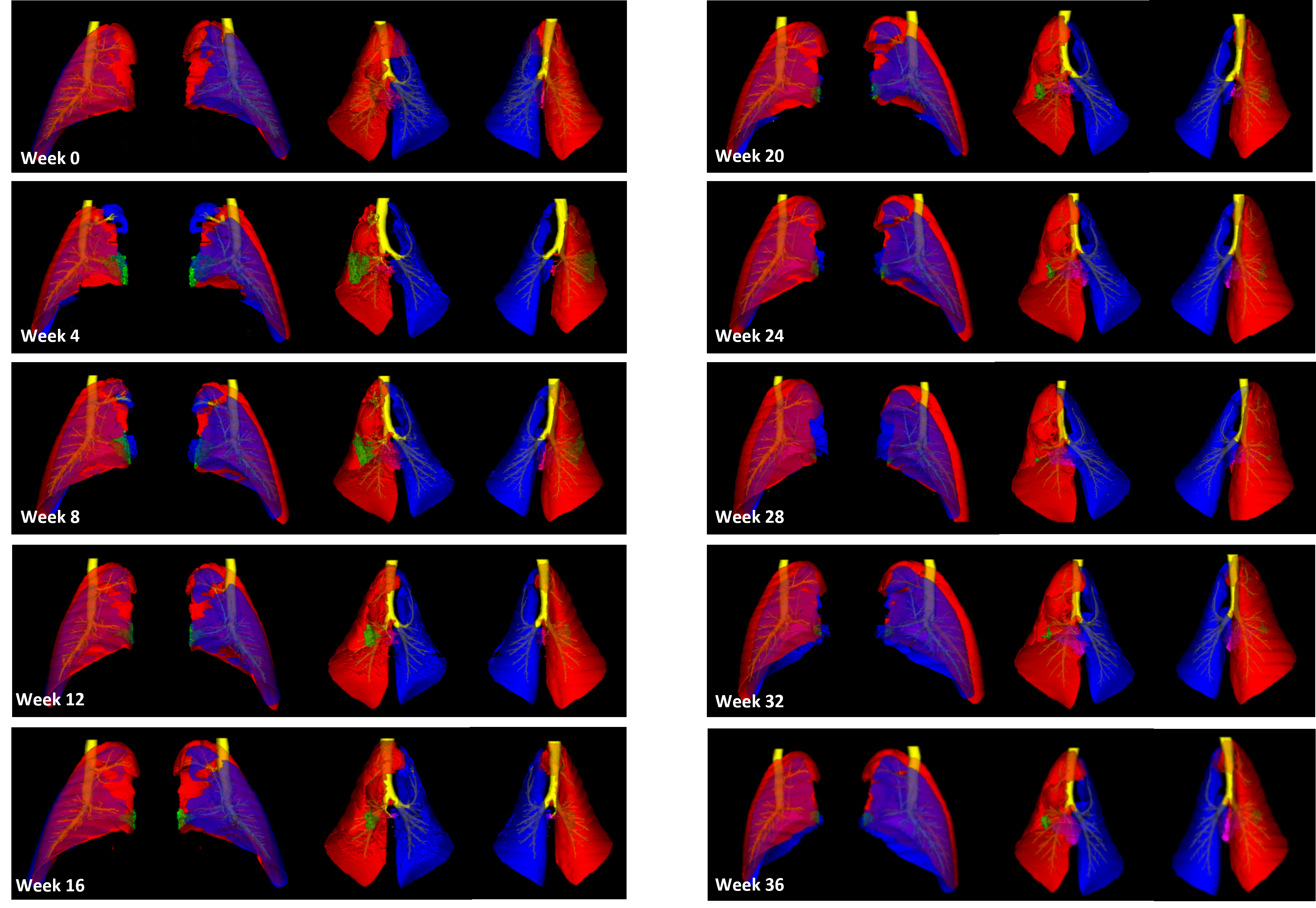

Supplement: Supplementary file 1 [file genes-15-01019-s001.zip › Supplementary Figure S9.tif]
